# Glacial vicariance drives phylogeographic diversification in the amphi-boreal kelp *Saccharina latissima*

**DOI:** 10.1038/s41598-018-19620-7

**Published:** 2018-01-18

**Authors:** João Neiva, Cristina Paulino, Mette M. Nielsen, Dorte Krause-Jensen, Gary W. Saunders, Jorge Assis, Ignacio Bárbara, Éric Tamigneaux, Licínia Gouveia, Tânia Aires, Núria Marbà, Annette Bruhn, Gareth A. Pearson, Ester A. Serrão

**Affiliations:** 1 CCMAR- Centro de Ciências do Mar, Universidade do Algarve, Faro, Portugal; 2 Department of Bioscience, Aarhus University, Silkeborg, Denmark; 3 Arctic Research Centre, Aarhus University, Aarhus, Denmark; 4 Centre for Environmental and Molecular Algal Research, University of New Brunswick, Fredericton, Canada; 5 Biocost Research Group, Universidade de A Coruña, A Coruña, Spain; 6 NSERC Industrial Research Chair for Colleges in Marine Macroalgae, Cégep de la Gaspésie et des Îles, Grande-Rivière, Québec, Canada; 7 Department of Global Change Research, IMEDEA (CSIC-UIB), Esporles, Spain

## Abstract

Glacial vicariance is regarded as one of the most prevalent drivers of phylogeographic structure and speciation among high-latitude organisms, but direct links between ice advances and range fragmentation have been more difficult to establish in marine than in terrestrial systems. Here we investigate the evolution of largely disjunct (and potentially reproductively isolated) phylogeographic lineages within the amphi-boreal kelp *Saccharina latissima* s. l. Using molecular data (COI, microsatellites) we confirm that *S. latissima* comprises also the NE Pacific *S. cichorioides* complex and is composed of divergent lineages with limited range overlap and genetic admixture. Only a few genetic hybrids were detected throughout a Canadian Arctic/NW Greenland contact zone. The degree of genetic differentiation and sympatric isolation of phylogroups suggest that *S. latissima* s. l. represents a complex of incipient species. Phylogroup distributions compared with paleo-environmental reconstructions of the cryosphere further suggest that diversification within *S. latissima* results from chronic glacial isolation in disjunct persistence areas intercalated with ephemeral interglacial poleward expansions and admixture at high-latitude (Arctic) contact zones. This study thus supports a role for glaciations not just in redistributing pre-existing marine lineages but also as a speciation pump across multi-glacial cycles for marine organisms otherwise exhibiting cosmopolite amphi-boreal distributions.

## Introduction

Glacial vicariance, the geographical separation of species into regional populations associated with glacial conditions, is one of the most pervasive and recurrent drivers of phylogeographic structure and speciation^1^. Genetic differences accumulate in isolated gene-pools and thus may eventually lead to new genomic recombinations in subsequent contact events or reproductive isolation. High-latitude species have been particularly prone to undergo such processes as a consequence of the cyclic growth and decay of the ice-sheets during the Quaternary ice-age (2.5 Ma ago to present)^2^. Continental-scale changes in regional temperature and oceanographic circulation regimes, among others, have resulted in important latitudinal shifts in the distribution and connectivity of biodiversity. For most high-latitude species in the northern hemisphere, glacial maxima, such as the last glacial maximum (LGM, ca. 26.5–19 ka)^3^, would have been characterized by more southerly, and often more fragmented, geographical distributions, such as the disconnected southern European peninsular refugia^4^. Likewise, with each ice advance and retreat, shallow coastal areas were also alternately exposed and submerged^5^, opening and closing dispersal corridors for marine and terrestrial biota such as the glacial Beringia land bridge (currently Bering Strait).

Speciation, the evolution of reproductive isolation leading to the formation of new biological species, can be an extended process spanning multiple climatic shifts. Diverging gene pools thus only become evolutionarily relevant from a speciation perspective if they survive and retain their integrity (i.e. do not fully merge) in the longer-term. Low-dispersal species are often subdivided in phylogeographic groups that are largely parapatric and presumably formed when isolated subpopulations expanded and contacted, a common process post-glacially^6–10^. Even when species ranges become more continuous, gene-flow can continue restricted beyond secondary contact/hybrid zones, so that pre-existing genetic differences are preserved, at least in original refugial areas. This can be due to limited dispersal and/or density-barrier effects^10^, or evolving post-zygotic (e.g. Dobzhansky-Muller) incompatibilities^11^. Such a composite mode of divergence and speciation, characterized by cyclic regional isolation and accumulation of genomic differences that are conserved beyond narrow and often geologically temporary interglacial contact/ hybrid zones, now seems pervasive among low-dispersal terrestrial taxa^4,7,12,13^.

Links between glacial/sea-level dynamics, allopatric divergence and speciation have been more challenging to document in the marine realm^14–16^, where often dispersal barriers are elusive, detailed fossil records unknown, and glacial histories obscured by intrinsic high rates of dispersal. Still, approaches including phylogeography and niche modelling have revealed effects of past climatic shifts and changes in the geographic distribution of marine organisms and/or of their lineages. Northern extirpations, climatic refugia (including northern refugia) and post-glacial contact/hybrid zones have been identified across the world oceans, and several pathways of poleward post-glacial expansions established^17–22^. However, explicit biogeographical settings of marine glacial vicariance, and how they might have actually contributed to generate new marine biodiversity in the long-term, have seldom been identified (but see^21,23^).

The sugar kelp *Saccharina latissima sensu lato* (s.l.) was recently shown to be composed of three lineages of uncertain biological status, morphologically cryptic but genetically distinct [based on the barcoding mitochondrial cytochrome c oxidase gene (COI, COX1 or CO1, <700 bp]^24^. Phylogroup divergence and distributions, matching the NE Pacific, NW Atlantic and NE Atlantic shores (with some overlap in the Canadian Arctic), led these authors to hypothesize that *S. latissima* s.l. could correspond to a complex of incipient species, presumably resulting from repeated cycles of glacial isolation and interglacial migration and contact. Internal transcribed spacer (ITS, located between the small and large subunits of the nuclear ribosomal RNA genes) data, however, did not recover the same geographic clustering, and many NW Atlantic individuals exhibited ambiguous base calls (i.e. heterozygosity) suggestive of re-established gene-flow. Because incomplete lineage sorting and/or concerted evolution of ITS sequences could also explain low phylogeographic signals and resemble admixture, the status of these mtDNA phylogroups remained unresolved.

At a higher level, the genetic circumscription of *S. latissima* s.l. itself is also probably incomplete. Morpho-species delimitation within *Saccharina*, and more generally the taxonomy of Laminariales, is afflicted by extensive intra-specific plasticity (ontogenic, ecological, etc.) and/or convergence^25–27^. The genus, for instance, was only recently separated from *Laminaria*^28^, and now includes several taxa formerly placed in e.g. *Hedophyllum*, *Kjellmaniella*, *Cymathaere*^28–30^. *S. latissima* has been reported but not genetically confirmed from the NW Pacific^31^, where a range of other putative endemic congeners also occur. Recently, the COI gene has been successfully employed as a standardized DNA barcode marker in a range of kelp genera, including *Saccharina*^24,32–35^. Besides the Atlantic phylogroups of *S. latissima* s.l., COI has clarified the identity and geographical ranges of Pacific *S. bongardiana*, *S. nigripes* and cryptic *S. druehlii*^24,33,34^. While other morpho-taxa from the NW Pacific (e.g. *S. cichorioides* complex) appear to be genetically very similar and possibly conspecific to *S. latissima* s. l.^36,37^, they have not been examined for this same discriminant marker. Definite species boundaries and genealogic relationships of *S. latissima* s. l. remain therefore unclear^29^.

In this study, based on an extended panel of populations and the integration of environmental, COI barcoding and microsatellite genotypic data, we address several related questions regarding the identity and biogeography of *S. latissima* s. l. Specifically, we aimed to clarify 1) the current taxonomic circumscription and global range of *S. latissima* s. l. and 2) the validity, genetic integrity (reproductive isolation in sympatry) and biogeographical history of previously described mtDNA phylogroups. We confirmed the presence of *S. latissima* in the NW Pacific, where specimens had previously been classified as distinct species, and mapped with unprecedented scale and resolution the complex distribution of phylogroups in the Arctic/Atlantic. There, we inferred the sequence of post-glacial poleward migration of phylogroups from disjunct glacial ranges based on phylogeographic and palaeo-environmental evidence. This and their general isolation along a modern secondary contact zone support the role of glacial vicariance as a driver of marine allopatric divergence and speciation, a role that is often hypothesized but poorly documented with good empirical evidence. These findings are particularly relevant for the understanding of the evolution and biogeography of amphi-boreal species complexes and other high-latitude marine biota most directly affected by periodic glacial advances.

## Results

### COI data

**Figure 1:**
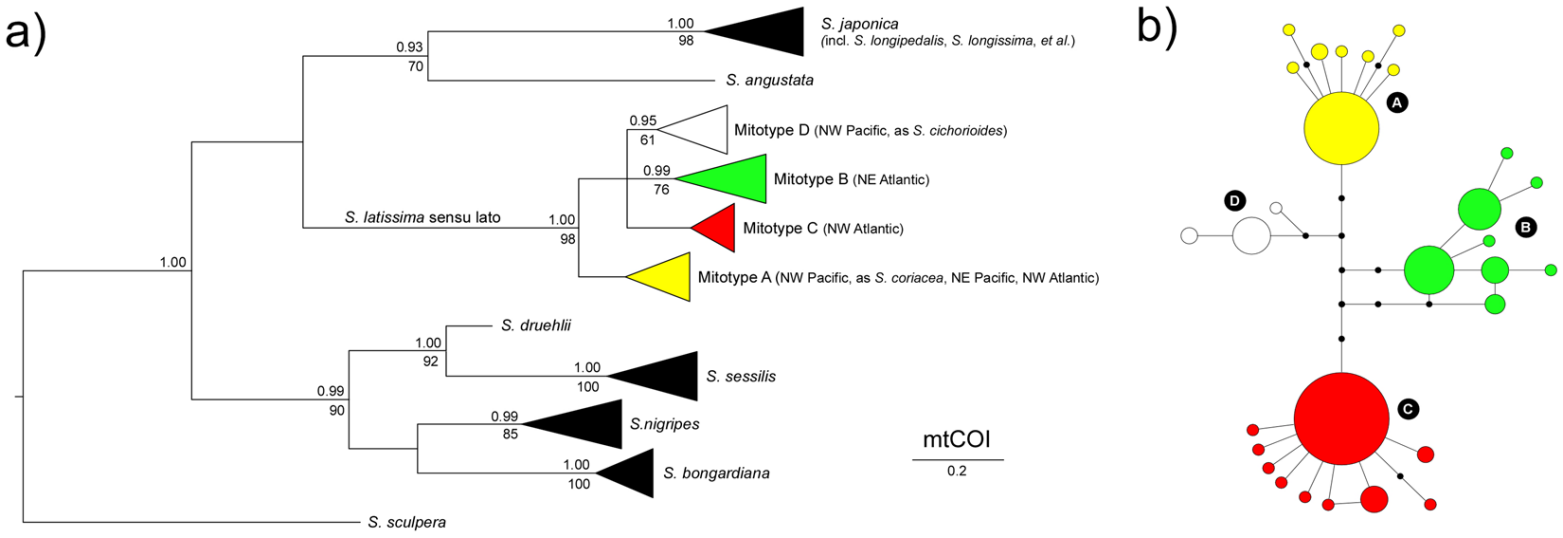
Genealogic relationships within *Saccharina* based on mtCOI sequence data. (**a**) Bayesian 50% majority-rule consensus tree based on 62 unique sequences. Numbers above and below the branches are Bayesian posterior probabilities (>0.90) and maximum likelihood bootstrap support values (>60), respectively. Horizontal triangles represent collapsed branches, with length (horizontal) representing the distance from the branches’ common node to the tip of the longest branch, and height (vertical) scaled to the number of (unique) sequences collapsed. (**b**) COI haplotype network of *S. latissima* sensu lato, showing the four (A–D) inferred mitotypes. Haplotypes are represented by circles sized to their frequency. Black dots represent inferred, unsampled haplotypes.

The global alignment of COI sequences of *Saccharina* spp. was 658 bp long and included 225 sequences retrieved from Genbank (Table S1), plus 51 newly generated sequences of Atlantic *S. latissima* s. l. from the eastern Canada, Greenland and Europe. The final alignment used in phylogenetic analyses consisted of a subset of 62 unique sequences (most COI sequences were identical within species/phylogroups, demonstrating the usefulness of this locus for barcoding purposes), and the best-fit nucleotide substitution model was determined to be the Hasegawa-Kishino-Yano^38^ (HKY + G; different transition/transversion rates, unequal base frequencies) with gamma distributed rate variation among sites. Recovered ML and Bayesian trees revealed three well supported clades containing a total of 11 taxa: 1) *S. japonica* s. l. (comprising many genetically indistinguishable taxa) and *S. angustata*, 2) *S. latissima* s. l. (including *S. cichorioides* and *S. coriacea*, see below), and 3) *S. bongardiana*, *S. nigripes*, *S. sessilis* and *S. druehlii* (Fig. 1a, see also Table S1 for original species designations). Low phylogenetic signal of this fragment of COI did not allow further resolution of the tree.

**Figure 2:**
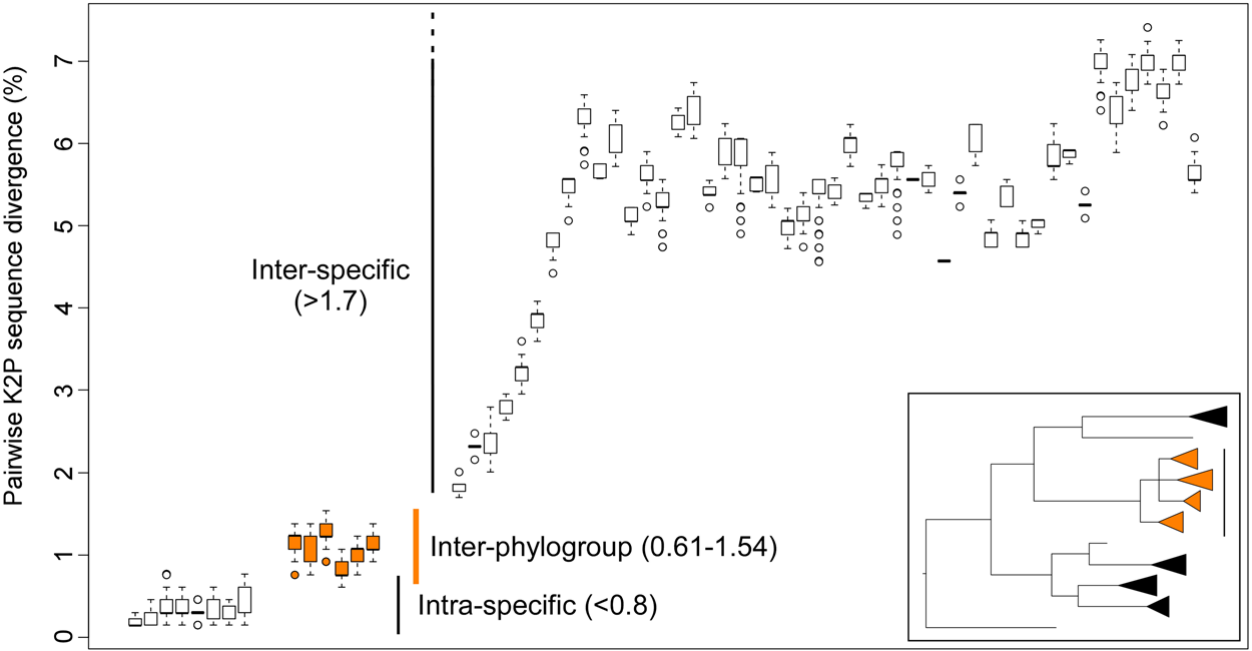
Range of K2P sequence divergences (%) within recovered genetic entities of *Saccharina* spp. Boxplots are grouped according to the type of pairwise comparison: intra-specific (left, intra-phylogroup in the case of *S. latissima* s.l.), inter-phylogroup (mid graph, in orange) and inter-specific (right).

**Figure 3:**
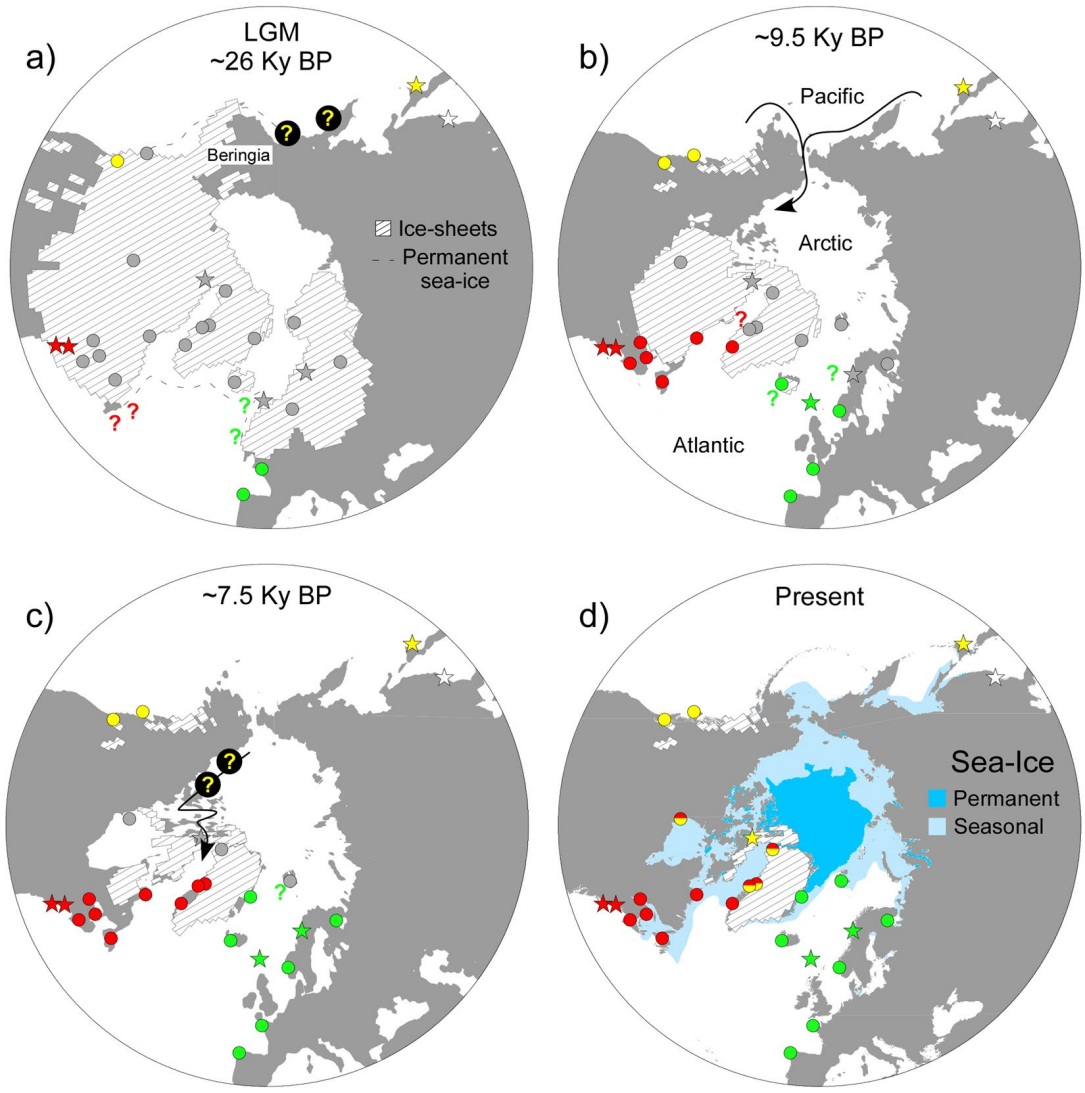
Glacial vicariance and post-glacial secondary contact of Atlantic phylogroups of *S. latissima*. The hypothetical glacial ranges and re-distribution of mtCOI phylogroups since the LGM (as inferred from indirect phylogeographic and paleoenvironmental data) are illustrated with a sequence of time points starting with (**a**) maximum ice-sheet size, (**b**) the opening of the Bering Strait, (**c**) the collapse of the Laurentide ice-sheet and the transgression of the Hudson Bay and (**d**) the present. Phylogroups are coloured as in Fig. 1 (COI-A: yellow, COI-B: light green; COI-C: red, COI-D: white). Sites that were also genotyped for microsatellite markers are marked as circles, otherwise stars. Sites in grey correspond to inhospitable areas (emerged, or under ice-sheets and perennial sea-ice) or beyond ice barriers. Maps were generated with QGIS 2.17 (http://qgis.osgeo.org) using modelled ice-sheet and land extent data^54^.

*S. latissima* s. l. was subdivided in four differentiated phylogroups, separated by 4–6 fixed mutations (Fig. 1b). These correspond to the three mitotypes (Pacific, European and Atlantic) previously identified by McDevit and Saunders^24^, herein designated phylogroups A, B and C, respectively, plus a fourth one, originally identified as *S. cichorioides* (here phylogroup D). Maximum K2P divergence within recognized species was typically below 0.8%, whereas interspecific divergences ranged between 1.7 (closely related *S. sessilis* & *S. druehlii*) and 7.26% (Fig. 2, Table S2). *S. latissima* s. l. exhibited atypically high (maximum) intra-specific divergence (K2P < 1.54%), with sequence divergences between different phylogroups ranging from 0.61–1.07% (A vs D) to 0.92–1.54% (A vs C). Phylogroups exhibited contrasting distributions on the Atlantic and the Pacific oceans (Fig. 3, last panel). Phylogroup A was detected in both NW (Japan, as *S. coriacea*) and NE (British Columbia) Pacific and also in the colder areas of the NW Atlantic (W Greenland and Hudson Bay). The remaining phylogroups were restricted to a single biogeographic region – B and C throughout NE and NW Atlantic, respectively, and D (sampled as *S. cichorioides*) was genetically confirmed only from Russia’s Pacific region of Primorye.

### Microsatellite data

**Figure 4:**
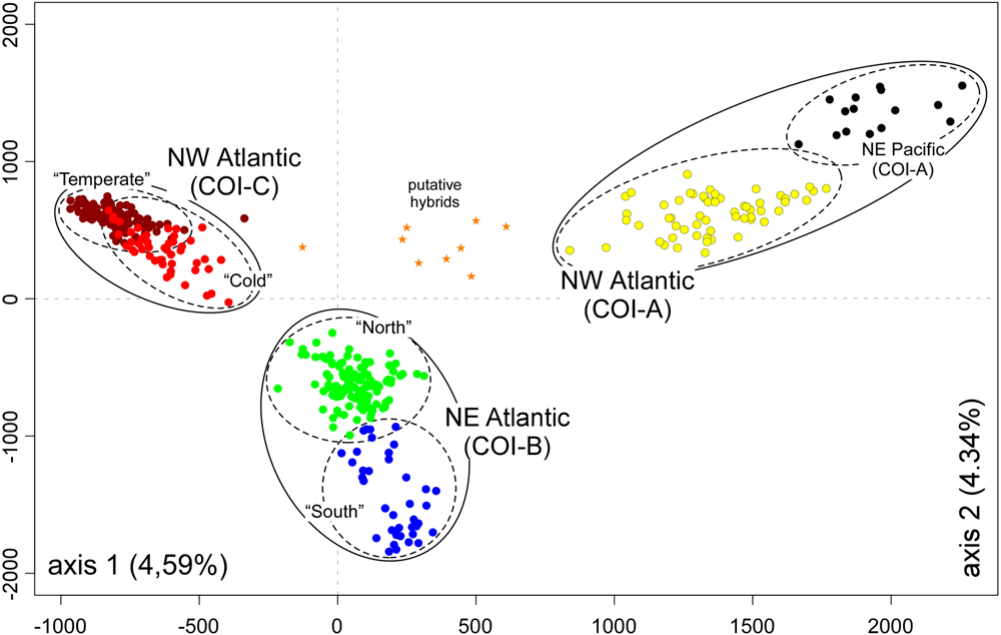
FCA plot based on all individual multilocus genotypes of *S. latissima* s. l. Solid lines denote mtDNA phylogroups (note the correspondence with microsatellite-based genotypic clusters) and dashed lines further geographic sub-divisions inferred with genotypic data alone. All putative hybrids were detected in NW Greenland (NW Atlantic) within mixed COI-A/COI-C populations. The “temperate” COI-C outlier could result from backcrossing, genotyping error, or other.

**Figure 5:**
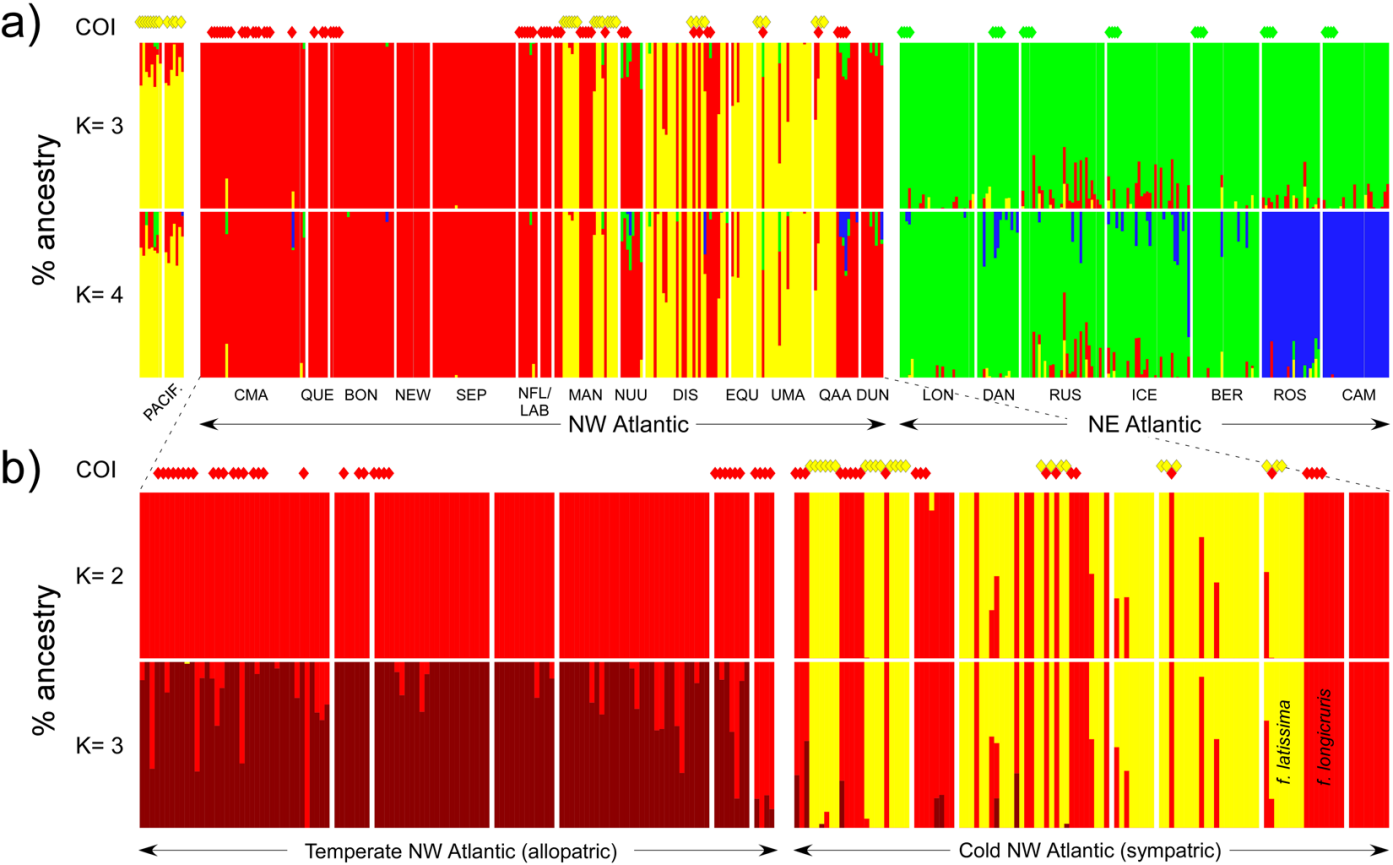
Population structure of *Saccharina latissima* s. l. based on LEA analyses. (**a**) Percentage ancestry of each genotyped individual (vertical bars) from the northeast Pacific and north Atlantic/Arctic, when the number of ancestral genetic clusters (k) is set to k = 3 (top) and k = 4 (bottom). (**b**) Percentage ancestry of individuals from the northwest Atlantic and northwest Greenland, for k = 2 (top) and k = 3 (bottom). COI phylogroups are shown on top of each plot for comparison. Population codes as in Table 1, colour codes as in previous figures.

Genetic diversity within populations was very variable but highest in NE Atlantic (Table 1), and regional differences in allelic composition were readily apparent (Fig. S1). The genotypic clusters recovered matched remarkably well the COI phylogroups, as illustrated in the FCA plot (Fig. 4). Shallower subdivisions were also apparent within phylogroups, e.g. NE Pacific vs NW Atlantic populations of phylogroup A, or “southern” (Iberia and Brittany) and “northern” (remaining) populations of phylogroup B. LEA admixture analyses also recognized 3 (or 4) stable genotypic clusters in the whole data-set (Fig. S2a), matching these same groups (Fig. 5a). When analysed separately, 2 (or 3) stable genotypic clusters were detected in the NW Atlantic (Fig. S2b), again matching remarkably well A and C phylogroups, and the mild separation of the latter in a temperate (allopatric) and a colder (sympatric) subpopulations (Fig. 5b). Both FCA and admixture plots revealed intermediate A/C genotypes along their sympatric cold NW Atlantic range (Figs 4 and 5b). In the temperate NW Atlantic (phylogroup C), individuals exhibiting *longicruris* (long hollow stipes) and *latissima* (short solid stipes) morphologies were genetically indistinguishable based on both COI and microsatellite markers. Conversely, in Manitoba and W Greenland, many individuals belonging to phylogroup C were originally collected as *S. longicruris*, whereas those belonging to phylogroup A were always collected as *S. latissima* (see also McDevit and Saunders^24^). This pattern is well illustrated in sympatric individuals from QAA. The highest heterozygote deficiencies were detected in these mixed A/C populations (Table 1).

**Table 1:** Geographic origin and genetic diversity of populations of *Saccharina latissima* s.l.

	Population, Country	Code	Lat	Lon	COI	n	A	A_(15)_	H_E_	H_O_	F_IS_
Pac	“Vancouver Island”, CA	BCV	49.0280	−124.0487	A	8	2.9	NA	0.375	0.326	0.140
	“Haida Gwaii”, CA	BCH	52.5801	−131.4913	A	7	2.7	NA	0.324	0.312	0.040
NW Atlantic	“Canadian Maritimes”, CA^1^	CMA	45.0523	−65.2004	C	38	5.1	3.85 ± 0.24	0.332	0.256	0.231*
	“Quebec”, CA	QUE	48.3480	−69.3990	C	7	3.1	NA	0.412	0.413	−0.002
	Ile Bonaventure, CA	BON	48.5058	−64.1726	C	23	3.8	3.71 ± 0.16	0.454	0.466	−0.027
	Newport, CA	NEW	48.2912	−64.7159		12	4.0	NA	0.448	0.431	0.040
	Sept-Iles, CA	SEP	50.1772	−66.3639		30	3.9	3.28 ± 0.16	0.382	0.364	0.048
	“Newfoundland”, CA	NFL	48.6513	−56.3193	C	7	2.8	NA	0.401	0.319	0.258
	“Labrador”, CA	LAB	58.9318	−63.2228	C	4	1.3	NA	0.140	0.146	−0.050
	“Manitoba”, CA	MAN	58.7848	−94.0606	A, C	23	3.8	3.53 ± 0.12	0.563	0.274	0.519*
	Nuuk, GL	NUU	64.1720	−51.7239	C	8	3.4	NA	0.461	0.441	0.047
	Disko, GL	DIS	69.2458	−53.5277	A, C	30	5.8	4.87 ± 0.24	0.644	0.433	0.331*
	Eqip Sermia, GL	EQU	69.2199	−51.1252		8	3.3	NA	0.464	0.313	0.342*
	Uummannaq, GL	UMA	70.6746	−52.1310	A, C	20	3.9	3.81 ± 0.16	0.475	0.381	0.202*
	Qaanaq (lati type), GL^2^	QAS	77.4661	−69.2438	A,C	8	3.0	NA	0.417	0.313	0.277*
	Qaanaq (long type), GL^2^	QAL			C	8	2.0	NA	0.266	0.229	0.147
	Dundas, GL	DUN	76.5412	−68.8428		8	2.2	NA	0.333	0.296	0.119
NE Atlantic	Longyearbyen, SJ	LON	78.2232	15.6267	B	27	5.3	4.35 ± 0.21	0.472	0.472	−0.001
	Daneborg, GL	DAN	74.2991	−20.2292	B	15	3.7	3.83 ± 0	0.434	0.393	0.097
	White Sea, RU	RUS	66.3414	33.7135	B	30	8.3	6.68 ± 0.23	0.632	0.583	0.078*
	Iceland, IS	ICE	64.1540	−21.8514	B	30	7.3	5.63 ± 0.26	0.618	0.530	0.146*
	Bergen, NO	BER	60.2697	5.2222	B	24	6.3	5.42 ± 0.28	0.608	0.564	0.075*
	Roscoff, FR	ROS	48.7287	−3.9869	B	21	6.1	5.56 ± 0.20	0.541	0.532	0.018
	Camariñas, ES	CAM	43.1219	−9.1927	B	24	4.5	4.01 ± 0.17	0.412	0.431	−0.047

CA: Canada; GL: Greenland; SJ: Svalbard; RU: Russia; IS: Iceland; NO: Norway; FR: France; ES: Spain; *Lat/Lon in decimal degrees, averaged in artificial populations (in brackets; when individuals were collected at multiple sites and dates inside the general area).^1^“Canadian Maritimes” includes samples from New Brunswick, Nova Scotia and Prince Edward Island. ^2^Lati type = “*S. latissima* morphotype”; Long type = “*S. longicruris* morphotype”. COI: haplogroups sampled; n: individuals genotyped; A: mean allelic richness; A′: standardized number of private alleles; H_E_: Nei’s gene diversity; H_o_: observed heterozygosity; F_IS_: multi-locus inbreeding coefficient (*if significant, 1000 permutations).

**Figure 6:**
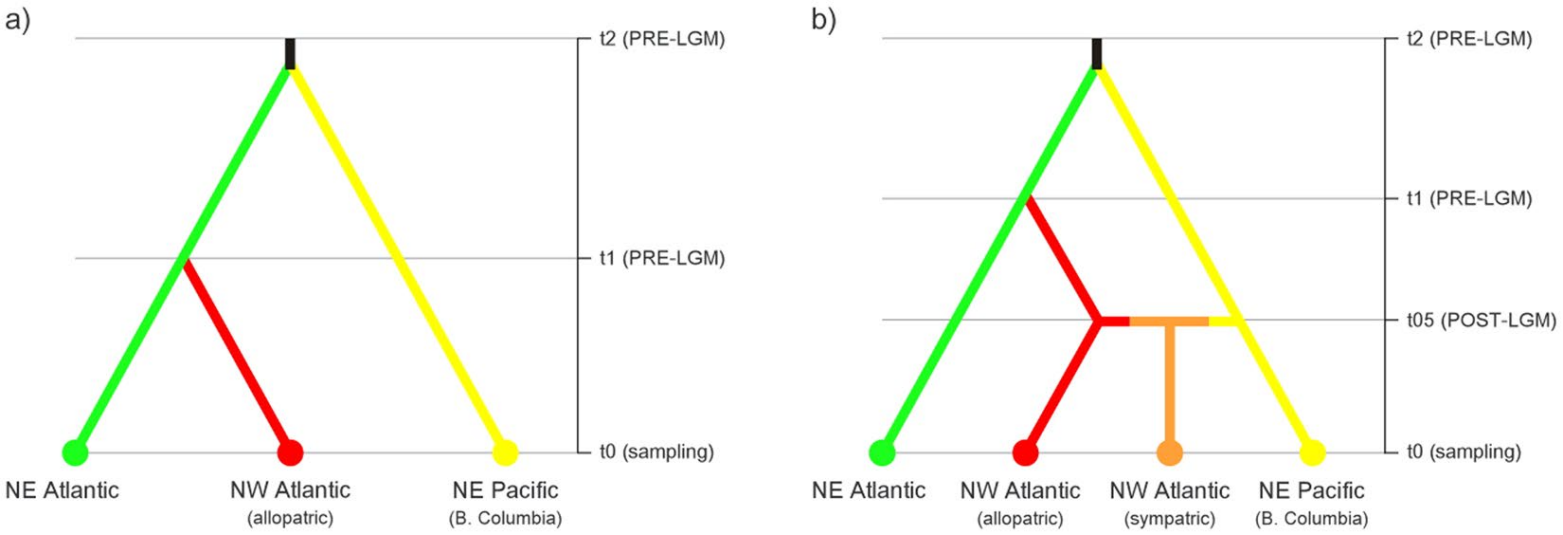
Diagram of the most likely demographic scenarios chosen by the (**a**) first and (**b**) second hierarchal levels of ABC analyses.

The first hierarchal analysis of ABC, testing 18 scenarios regarding the topology of population ancestry, revealed that the Pacific and Atlantic gene-pools diverged from a common ancestor ~198,000 generations ago, with NE and (allopatric) NW Atlantic *S. latissima* diverging much latter, around ~90,000 generations ago (scenario 5; Figs 6a and S3a; Table S3). Unsurprisingly, the second hierarchal ABC analyses identified the Pacific (A) and (allopatric) NW Atlantic (C) gene-pools as the main putative sources of the current mixed region of W Greenland and Hudson Bay (scenario 6; Figs 6b and S3b). This contact zone was estimated to have formed ~3,550 generations ago, i.e., post-glacially (Table S4). Error rates were estimated as 0.124 and 0.045, for the first and second hierarchal levels of ABC, respectively.

## Discussion

Molecular data have contributed greatly to resolve *Saccharina* within the Laminariales and to refine morpho-species boundaries within the genus. Despite these advances, a comprehensive and stable taxonomical treatment of *Saccharina* has yet to be produced. Phylogenetic analyses revealed negligible COI divergence between *S. japonica* and a multitude of named and unnamed taxa from the NW Pacific, confirming that many morpho-taxa from the NW Pacific correspond to mere varieties of this highly plastic species^37,39^ (see also COI-based^37,40^ and ITS/rubisco-based^28,30,36^ studies). More relevant to our study, COI data were incompatible with the current taxonomic interpretation of *S. latissima*, with interesting systematic and biogeographic implications.

Thus far, *S. latissima* was known to comprise three phylogroups with contrasting distributions in the NE Pacific and N Atlantic^24^. However, the COI tree revealed that *Saccharina* samples from Hokkaido (Japan) and Primorye (Russia), originally identified as *S. coriacea*^40^ and *S. cichorioides*^37^, respectively, were also embedded within *S. latissima*. Previous studies already showed very close evolutionary relationships between these taxa. For instance, Yotsukura *et al*.^36^ recovered nearly identical ITS1 and ITS2 sequences (only 1 bp indel) between *S. coriacea*, *S. cichorioides* and *S. yendoana* from Hokkaido and a western Canadian sample of *S. latissima*. These authors discussed the possibility that these taxa represented a single biological species, but recognized that low ITS resolution could mask biologically meaningful differences between closely related species, and thus that more data would be necessary to settle the issue. Likewise, Balakirev *et al*.^37^ also showed that her own samples of *S. cichorioides* and *S. coriacea* from Hokkaido (in our analyses corresponding to phylogroups D and A, respectively) exhibited identical ITS and plastid rbcLS sequences, and interpreted them as being conspecific.

Collectively, these data concur that *S. cichorioides* s. l. (sensu Selivanova *et al*.^41^, including also *S. coriacea*, *S. yendoana* and *S. sachalinensis*) and *S. latissima* s. l. (sensu McDevit and Saunders^24^, including also NW Atlantic *S. longicruris*) correspond to the same group of related phylogroups and thus that they should be formally synonymized. For simplicity, and given nomenclatural priority, we keep *S. latissima* s. l. when referring to this broader and now decidedly amphi-oceanic definition of the complex. The taxonomic confusion around this species complex (at least four cryptic phylogroups mismatched with many poorly supported morpho-taxa) and *S. japonica* (an over-classified plastic species) illustrates well the pitfalls of classic taxonomy, and beg for the genetic confirmation of other NW Pacific endemic taxa such as *S. gurjanovae*, *S. kurilensis* (=*Cymathaere japonica*), and the closely related (as assessed with ITS^36^ *S. gyrata* (=*Kjellmaniella gyrata*). Undeniably, COI barcoding probably represents the most cost-effective approach to verify their identities, and also the most sensible approach to survey new regional floras, at least when multiple or potentially new species/phylogroups may be present^42,43^.

The limited number of genetically confirmed individuals prevents the mapping of the general ranges of the phylogroups A and D in the Pacific, including establishing if the latter is truly endemic to the NW Pacific coast. A true Pacific phylogeography would require extensive surveys along the coastlines of Japan, Russia, USA and Canada, which were not a goal of this study. Taking into consideration the ecologic and morphologic plasticity of *S. latissima* throughout Japanese and Russian shores, underlying the current multitude of described morpho-taxa, and the very limited (Japanese Sea, British Columbia) or missing (e.g. Okhotsk, Bering and Chukchi Seas) genetic information for other Pacific regions, additional unaccounted diversity cannot be completely ruled out. By comparison, our knowledge concerning the identity and distribution of phylogroups in the Atlantic is now much more complete. Phylogroups B and C were confirmed to have disjunct distributions in NE (Iberia to Svalbard) and NW (Long Island Sound to northern Baffin Bay) sides of the Atlantic, as well as on each side of Greenland. The Atlantic distribution of phylogroup A was also extended in this study to include whole of W Greenland, where it was often observed in local sympatry with phylogroup C.

Intra-specific COI divergence within *S. latissima* s. l., as noted before^24^, is atypically high when compared to other kelp species, including widespread and morphologically plastic congeners such as *S. japonica*^39^ and *S. nigripes* (originally as S. *groenlandica*^24^). Multi-locus genotypic clusters matched remarkably well COI phylogroups, confirming that differences are genome-wide and constant throughout their ranges and not reflecting some single-gene idiosyncrasy. Shallower, intra- oceanic microsatellite structure was also apparent within phylogroups, but with much weaker phylogeographic signal. In the NE Atlantic (phylogroup B), a “southern” (Iberia plus Brittany) and a “northern” genotypic clusters were relatively well resolved, as was to some extent a similar southern/northern subdivision in the NW Atlantic (phylogroup C). Widespread phylogroup A also revealed mild (recovered only in the FCA) genotypic differences between Pacific and Atlantic samples that deserve further examination.

At least in the Atlantic, phylogroup divergences matching simultaneously unique nuclear backgrounds and biogeographic ranges, strongly suggest that these lineages have had largely independent evolutionary histories (see next section), and may potentially represent incipient species. Laboratory crosses between *S. latissima* originating from Vancouver (most likely phylogroup A), Halifax (sampled as *S. longicruris*, phylogroup C) and Roscoff/Helgoland (phylogroup B) show full inter-fertility and result in normal, viable hybrids^44^. A range of pre- and post-zygotic mechanisms may nevertheless influence real hybridization (and hybrid survival) rates in the wild, which may be challenging to assess directly. A few mixed populations were detected throughout the overlapping ranges of phylogroups A and C in western Greenland and Hudson Bay. These mixed populations exhibited the largest heterozygote deficiencies, suggesting that hybridization is not frequent. Indeed, individuals with intermediate genotypes (putative hybrids and backcrosses, <90% assignment to either A or C clusters in the admixture analyses) were detected in at least 4 of these populations, but in very low frequencies. In Manitoba, where A and C phylogroups were sampled with a 3/2 ratio, admixed genotypes were not even detected. While the nature of the biological isolation mechanisms that may be operating is not fully identified, it seems clear that rates of gene-flow following contact have been insufficient to compromise their integrity at both local and regional scales.

Globally, the available genetic data (phylogeography and apparent paucity of genetic hybrids in sympatry) is clearly compatible with the hypothesis of *S. latissima* s.l. corresponding to a complex of incipient species. Unfortunately, despite the sampling effort, an expected contact zone between NE (B) and NW (A & C) Atlantic phylogroups could not be located, making it impossible at present to determine their actual degree of isolation. Mixed populations may still occur along the southern tip of Greenland, as different phylogroups are established on each side of the island. Future sampling campaigns should target this area to look for potential mixed populations, and, if these eventually are found, to assess their degree of isolation. The same applies to the NW Pacific range, where phylogroup ranges, contact areas and reproductive isolation remain basically unassessed. It also remains to be assessed if there is any constant and unambiguous diagnostic character allowing field/laboratory identification of *S. latissima s. l*. or of any of its constituent phylogroups. A robust multi-locus phylogeny, additional morphological and phylogeographical data, and particularly detailed characterization of additional contact zones are likely to bring new insights on the full diversity and relationships of lineages within the species complex, and ultimately help determine their most adequate biological and taxonomic status.

Genomic differences take time to accumulate and typically require a significant degree of geographic isolation between diverging sub-populations. In general, dispersal barriers are perceived to be less absolute and more transient at geological time-scales in marine *vs* terrestrial ecosystems, and the environmental conditions promoting allopatric divergence are also expected to differ. For instance, marine analogs of terrestrial glacial refugia such as “disjunct southern peninsulas” are unexpected to form, and persist in the long-term, within south/north-oriented coastlines characterized by relatively continuous thermal gradients, habitat availability and species distributions^45^. This paradigm of glacial vicariance, however, may be useful to explain population structure of *S. latissima* and other cosmopolitan amphi-boreal species.

During glacial maxima, such as the LGM, the Arctic and much of the adjacent continents and oceans were covered with massive ice sheets, thick ice-shelves, and perennial sea-ice, making them virtually uninhabitable to most coastal species^46–49^. At the height of LGM, when the sea-level was ca. 120 m lower than today, the Eurasian ice-sheet spread from the Russian Severnaya Zemlya archipelago to the British Isles across the emerged Kara, Barents and North seas. In the Pacific, the modern Bering Sea provided a terrestrial passage (Beringia) between the Eurasian and American continents and supressed the modern connection between the Pacific and the Arctic oceans. The Laurentide ice-sheet also spread across the modern Hudson Bay and the Canadian Arctic Archipelago to connect with the Greenland ice-sheet, forming a second barrier preventing trans-Arctic exchanges between the Pacific and the NW Atlantic oceans.

Remarkably, the current distribution of phylogroup A in the Canadian Arctic and western Greenland coincides with one of these regions that were most heavily glaciated and inhospitable during the LGM. Its absence further south in more temperate regions of the NW Atlantic, like phylogroup C, implies that its Arctic/Atlantic range must have been colonized only post-glacially from a N Pacific source, a scenario supported by the ABC demographic analyses and previously documented for other unrelated organisms^43,50,51^. If so, regional diversity levels in the Atlantic would be expected to be depressed when compared to putative Pacific sources. Because only a few individuals from two temperate sites in the British Columbia (BCV, BCH) were available for analyses, the diversity of these populations could not be compared with presumably recently established Arctic/Atlantic populations. Detectable allelic differences between these and NW Atlantic populations (Fig. S1) nevertheless suggest that the latter were not directly involved in the putative trans-Arctic migration. Populations located closer to the Bering Strait are more likely candidates, but only comparable molecular data from this and other N Pacific regions can provide definite evidence for it. In any case, the apparently unidirectional migration is likely to reflect the dominant oceanographic flow (Pacific->Arctic->Atlantic)^52^ but matches also the pattern of deglaciation. The Bering Strait opened around 9.5 Ky BP, allowing the eastward colonization of the Arctic along the seasonally ice-free Chukchi and Beaufort Seas (Fig. 3a,b), well before the northwest passage across the Canadian Arctic Archipelago was (seasonally) resumed or the Hudson Bay started deglaciating/transgressing (<7.5 Ky BP^53,54^; Fig. 3c).

A similar trans-Arctic migration along the Eurasian Arctic, potentially leading to secondary contact with phylogroup B, would also seem a possibility, especially given the earlier retreat of the Eurasian Ice-sheet and the uninterrupted existence of seasonal ice-free shorelines throughout Siberia. However, the lack of records suggests that this long Arctic coastline may not be particularly suitable for *S. latissima*, which could hypothetically be caused by extensive discharge of freshwater and sediments from Siberian rivers reducing salinity and rocky substrate. Biodiversity in the Laptev Sea for instance is very depauperate when compared to both White/Barents and Chukchi seas, and most diatoms and fish there are characteristic of brackish waters^55,56^. For sessile species like *S. latissima* lacking planktonic dispersive stages, these large distributional gaps may act as a potent trans-Siberian dispersal barrier.

Lineages B and C are certainly native to the Atlantic, i.e. evolved and split there, possibly after a much older (pre-LGM) colonization event from the diversification centre of *Saccharina* in the Pacific. Their ranges were also severely constrained at high latitudes during former glacial maxima. Environmental reconstructions of the LGM place the seasonally ice-free boundaries as far south as the British Isles (NE) and New England (NW) (Fig. 3a). Consequences in terms of surviving biota were more severe in the NW vs NE Atlantic, due to a combination of lower availability of rocky shores and more compressed temperate envelopes^57^. While cold-adapted, plastic species like *S. latissima* may have endured during these periods they were much more physically separated and presumably much less if at all connected across the two sides of the Atlantic. Indeed, given that potential stepping-stone coastal habitats across mid-latitudes in the Atlantic are so rare and sparse, and the improbability of cross-oceanic migration (via drift) of reproductive sporophytes, gene-flow between glacial subpopulations was probably negligible. Because glacial conditions are typically lengthier (at least one order of magnitude) than inter-glacial conditions^58^, disjunct glacial ranges and smaller population sizes help explain the observed NE/NW Atlantic divergence. Also, regular contact and admixture during interglacials are likely to be largely irrelevant evolutionarily. This is because secondary contacts during post-glacial expansions tend to form predominantly at high latitudes [as presently observed in western (and potentially southern) Greenland], so that admixed populations are among those more prone to be erased in subsequent glacial advances. On the contrary, pure, southern populations will tend to persist across climatic shifts.

A scenario of glacial vicariance intercalated with ephemeral interglacial admixture would have cumulative genetic and ultimately reproductive effects across multiple glacial cycles. It represents, in our view, the best explanation for the remarkable diversification and modern phylogeographic structure of *S. latissima* s. l. in the northern hemisphere, where the species currently exhibits a rather continuous cosmopolitan distribution that might suggest that genetic admixture opportunities are prevalent over long-term isolation and differentiation. There are many other amphi-boreal, spatially-structured species complexes in the northern Hemisphere, although in contrast with kelps these are mostly high-dispersal fish and invertebrates, e.g.^50,59,60^). All this evidence from marine organisms with distinct dispersal means suggests that episodic waves of trans-Arctic and trans-Atlantic migration and chronic glacial vicariance probably represent rather general drivers of marine diversification.

## Conclusions

The over-classification of *S. japonica* and *S. latissima*/*S. cichorioides* illustrates well the potential problems arising from the absence of clear and consensual diagnostic taxonomic characters in widespread plastic taxa, and the importance of sensibly analysing and interpreting genetic data available from public databases, especially when the underlying taxonomy of the studied group is to some extent unreliable and/or field identifications (even by experts) potentially incorrect (see also Shen *et al*.^61^). The COI barcode provides a standardized, cost-effective first step for species assignments in *Saccharina* spp. and new and more robust insights are likely to emerge as data from additional species and populations accumulate. Future molecular surveys should prioritize the NW Pacific diversity hotspot, where many putative endemic species occur, and where the diversity and distribution of genetically confirmed *S. latissima* s. l. remains basically unassessed. Finally, fine-scale studies focusing on modern contact zones – e.g. integrating morphologic, spatial and genetic (microsatellite) data – are required to further evaluate the nature and strength of the putative isolating mechanisms at play in this complex.

Cold-adapted species like *S. latissima s. l*. are fascinating models to investigate marine demographic responses, resilience and diversification associated with the Pleistocene climatic shifts. We attributed the clear genomic and biogeographic differences detected within this cosmopolitan species to prolonged glacial isolation in disjunct persistence areas intercalated with interglacial expansions and limited admixture in ephemeral, high-latitude contact zones. It remains to be evaluated whether present biodiversity coincides with relevant functional differences that could be important from a conservation or cultivation perspective, particularly in a scenario of ongoing climatic change and ever increasing demand for marine products. More generally, this study shows that glaciations are not just relevant in terms of the redistribution of pre-existing marine lineages but may actually function as an allopatric speciation pump, across multi-glacial cycles, for widespread amphi-oceanic organisms.

## Material and Methods

### Saccharina latissima

The sugar kelp *Saccharina latissima* (Linnaeus) C.E. Lane, C. Mayes, Druehl & G.W. Saunders (=*Laminaria saccharina* (L.) Lamouroux) is an edible, short-lived perennial, canopy-forming brown seaweed (Laminariales, Ochrophyta) widely distributed (and also cultivated) in shallow-water habitats throughout much of the Northern Hemisphere. Its documented but only partially confirmed range extends from the pack-ice border in the high Arctic to cold-temperate latitudes on both sides of the Atlantic and the Pacific oceans^31^. This vast distribution is accompanied by extensive ecological, morphologic and physiological plasticity, as well as substantial phylogeographic structure^24,62–64^.

### Genetic circumscription of *S. latissima*

Phylogenetic relationships within *Saccharina* were reconstructed with Bayesian and Maximum Likelihood (ML) inference methods using the barcoding COI mitochondrial gene. COI data included all published sequences of adequate range available (Table S1), as well as new sequences of *S. latissima* s. l. from both sides of the Atlantic and the Arctic (primers in^32^). PCRs were performed in 20 μL total volume containing ±10 ng of template DNA, 1x buffer, 2 mM of MgCl_2_, 0.5 mM of dNTPs, 0.5 μM of each primer and 1 U of GoTaq G2 Flexi DNA Polymerase (Promega). PCRs were run in a ABI 2720 Thermal Cycler (Applied Biosystems) with an initial denaturation step (95 °C, 5 min), 35 cycles of 95 °C for 30 s, 50 °C for 45 s and 72 °C for 1 min, and a final extension step (72 °C, 10 min). Sequences were aligned, proofread and edited in GENEIOUS 4.8 (Biomatters; http://www.geneious.com). Nucleotide substitution models (using 3 substitution schemes) were compared with jModelTest 2^65^ and best-fit models selected based on the Akaike information criterion. Bayesian analyses were performed using MrBayes 3^66^. Two parallel Metropolis-coupled Markov chain Monte Carlo searches, each with four chains (3 ‘heated’), were run for 2 × 10^6^ generations, sampling trees and parameters every 100 generations. The number of substitution rates, among-site rate variation, and base frequency priors were set according to the substitution model selected, leaving the remaining options as default. Run length sufficiency was confirmed by inspecting the average standard deviation of split frequencies between runs and cold chains Log-likelihood stationarity. Based on the latter, 2 × 10^5^ generations (2000 trees) were discarded as burn-in. The remaining 18000 trees sampled were used to produce 50% majority-rule consensus trees and to calculate branch posterior probabilities. Maximum likelihood analyses were performed with PhyML 3^67^ using the ATGC bioinformatics platform (http://www.atgc-montpellier.fr/phyml/). Nodal support was calculated using 1000 bootstraps, and trees were rooted with *S. sculpera* (=*Kjellmaniella crassifolia* Miyabe 1902, see Yoon *et al*.^30^).

The genealogic relationships within *S. latissima* s. l. were further illustrated with a network calculated with Network 4.6 ^68^, using the median-joining (MJ) algorithm. Divergence within and between recovered taxa were estimated in MEGA 7^69^ using Kimura’s two-parameter (K2P, allowing different transition/transversions rates)^70^ sequence distances.

### Population structure of *S. latissima* s. l. in the Atlantic

Populations of *Saccharina latissima* s. l. were collected throughout both sides of the Atlantic, from northwest Iberia to Svalbard and northeast Greenland (NE) and Quebec to northwest Greenland (NW) (Table 1). At each site, tissue samples (2–4 cm^2^) from a variable number of individuals (8–30) were collected haphazardly, safeguarding at least one meter between consecutive samples. These were preserved dried in silica-gel crystals and genomic DNA was extracted using the Nucleospin 96 Plant II kit (Macherey-Nagel, Germany). This sampling was complemented with individuals from the NW Atlantic and NE Pacific (British Columbia) previously analysed by^24,33^. The latter were collected at multiple sites and dates, but for simplicity individuals were pooled in artificial “populations” matching their general geographical origin (Table 1, populations in brackets). To confirm genetic differences between phylogroups and detect potential admixture versus reproductive isolation), multi-locus genotypes were produced for all individuals by screening 12 microsatellite loci developed specifically for *S. latissima* as described in Paulino *et al*.^71^. Amplified fragments were run in an ABI PRISM 3130xl automated capillary sequencer (Applied Biosystems) at CCMAR, Portugal. Microsatellite alleles were manually scored in STRAND (Veterinary Genetics Laboratory, University of California, Davis; http://www.vgl.ucdavis.edu/STRand) using the 500 LIZ size standard (Applied Biosystems) and binned using the R package MsatAllele^72^.

Summary statistics of the microsatellite genetic diversity, including allele frequencies, mean allelic richness (A), standardized number of private alleles (A_n_), Nei’s gene diversity (H_E_), observed heterozygosity (H_O_) and inbreeding coefficients (F_IS_), were calculated with GENETIX 4.05^73^. Population structure within north Atlantic *S. latissima* s. l., including its concordance with recovered mtDNA phylogroups, was assessed using genotype-based approaches. Genotypic structure was assessed with a factorial correspondence analysis (FCA) implemented in Genetix 4.05^73^ and with admixture analyses implemented with the R package LEA^74^. The latter performs Structure-like analyses^75^ but uses a much faster algorithm. Individuals were analysed without any a priori population assignments. Each number of assumed populations (K, set sequentially from 1 to 10–15, depending on analyses) was run 20 times with default parameter values, to the exception of the alpha that was set to 100. The best subdivisions of Atlantic *S. latissima* were determined by inspecting the minimal cross-entropy curve and the inter-run stability of genetic clusters produced for each K.

The evolutionary and demographic history of Atlantic phylogroups of *S. latissima* s. l. was further reconstructed using Approximate Bayesian Computations (ABC) as implemented in DIYABC^76^. We compared competing scenarios in two hierarchical levels^77^ in order to (i) identify the putative topology of ancestry and timing of divergence between the main phylogeographic groups identified (Fig. S4; NE Pacific, allopatric NW Atlantic and NE Atlantic, see results), and (ii) estimate the timing of secondary contact of divergent gene-pools across the NW Atlantic contact zone (Fig. S2b; previous groupings plus sympatric NW Atlantic, see results). We ran 10^6^ simulations per scenario to produce a set of pseudo-observed datasets (PODs). Effective population sizes and parameters for the Generalized Stepwise Mutation model were set to the program default^77,78^, while time of population divergence (in generations) were defined as uniform within pre- and post-LGM periods. Prior distributions for admixture rates were set between 0.001 and 0.999 (see Tables S5 and S6 for model parameters of each hierarchical level). The posterior probability of each scenario was inferred with a logistic regression performed on the 0.01 proportion of PODs closest to the empirical data^77–79^. Confidence in selected scenarios was evaluated by a Monte Carlo estimates of true and false allocations^78^.

### Availability of data and materials

The single new COI sequence was deposited in Genbank under accession number MF288586. Genotypic data were deposited in Figshare repository@ https://figshare.com/s/5a8fae0032a0148211a3.

## Electronic supplementary material

Supplementary files
