# Supplementary material for: Glacial vicariance drives phylogeographic diversification in the amphi-boreal kelp Saccharina latissima
Source: Sci Rep. 2018 Jan 18;8:1112. doi: 10.1038/s41598-018-19620-7 (PMC5773594; doi:10.1038/s41598-018-19620-7)
Supplement: Supplementary file 1 — Supplementary files [file 41598_2018_19620_MOESM1_ESM.pdf]

SUPPLEMENTARY FILES

Glacial vicariance drives incipient speciation in the amphi-boreal kelp *Saccharina latissima*

João Neiva, Cristina Paulino, Mette M. Nielsen, Dorte Krause-Jensen, Gary W. Saunders, Jorge Assis, Ignacio Bárbara, Éric Tamigneaux, Licínia Gouveia, Tânia Aires, Núria Marbà, Annette Bruhn, Gareth A. Pearson, Ester A. Serrão

Table S1. Published sources of mtCOI sequences of *Saccharina* spp. used in the phylogenetic analyses. Original species designations ([a] Genbank, [b] Journals), accession numbers, geographical origins and bibliographical sources are provided for each recovered genetic entity.

Putative species	Deposited[a]/published[b] as	Genbank accession(s)	Geographic origin	Source
S. sculpera	*S. sculpera/ Kjellmaniella crassifolia*	NC_029206 | KR350664	NW Pacific: unspecified	1
S. angustata	*S. angustata*	NC_013473 | AP011498	NW Pacific: Japan (Hokkaido)	2
S. japonica	*S. japonica*	NC_013476 | AP011493	NW Pacific: Japan (Hokkaido)	2
	S. religiosa/ S. japonica var. religiosa	NC_013477 | AP011494		
	S. ochotensis/ S. japonica var. ochotensis	NC_013478 | AP011495		
	S. diabolica/ S. japonica var. diabolica	NC_013482 | AP011496		
	S. longipedalis	NC_013484 | AP011497		
	S. longissima	NC_021640 | JN099684		3
	Saccharina sp. ye-C5	KT336421		4
	Saccharina sp. ye F	KP058499	NW Pacific: Russia (Sakhalin)	5
	Saccharina sp. ye-G	KM657964		6
	Saccharina sp. ye-W	KR056087		7
	Saccharina japonica, incl. var. longipes	JN873222-JN873238 (17)	NW Pacific: Russia (Primorsk)	8
	Saccharina sp. ye-C2-2/ S. sp. ye-C2	KT336420	NW Pacific: China (Liaoning)	9
	Saccharina sp. ye-C12	NC_028211| KT428594	NW Pacific: China (Qingdao)	10
	Saccharina sp. ye-C6	KT271760	NW Pacific: unspecified	11
	Saccharina sp. ye-B	NC_028192 | KT356873	unspecified	unpublished
	S. japonica	AB775228	NW Pacific: unspecified	12
	S. japonica, incl. varieties	KT963115-KT963144 (30)	NW Pacific: Russia (Sakhalin) to China (Shandong)	13
S. nigripes	*S. groenlandica/ S. bongardiana*	FJ409194-FJ409198 (5)	NE Pacific: Canada (B. Columbia)	14
	S. groenlandica	GU097731-GU097749 (19)	NE Pacific: Canada (B. Columbia); NW Atlantic: Canada (Hudson Bay, Canadian Maritimes)	15
S. bongardiana	*S. bongardiana*	GU097727-GU097730 (4)	NW Pacific: Russia (Kamchatka)	15
S. druehlii	*Saccharina sp. GWS-2014/ S. druehlii*	KJ960273	NE Pacific: Canada (British Columbia)	16
S. sessilis	*S. sessilis*	KJ960277,KJ960268, KJ960263,KJ960250, KJ960241 (5)	NE Pacific: Canada (British Columbia)	16
		FJ409205-FJ409208 (4)		14
		GU097833-GU097834 (2)		15
		EF218846		17
S. latissima A	*S. coriácea*	NC_013475 | AP011499	NW Pacific: Japan (Hokkaido)	2
	S. latissima	LT546291	NW Atlantic: Canada (northern Baffin Island)	18
	S. latissima (Pacific mitotype)	FJ409199-FJ409204 (6)	NE Pacific: Canada (B. Columbia); NW Atlantic: Canada (Hudson Bay)	14
		GU097754-GU097760, GU097762, GU097787, GU097789, GU097790, GU097792, GU097793, GU097797, GU097800, GU097812, GU097816, GU097819 (18)		15
S. latissima B	*S. latissima strain ye-C14*	NC_026108 | KM675818	NE Atlantic: Germany	19
	S. latissima	EU681420	NE Atlantic: France	20
		LN877849	NE Atlantic: Norway	unpublished
	S. latissima (European mitotype)	GU097750-53, GU097786, GU097803 (6)	NE Atlantic: Ireland, Faroes	15
S. latissima C	*S. latissima* (Atlantic mitotype)	GU097761, GU097763-85, GU097788, GU097791, GU097794-96, GU097798-99, GU097801, GU097802, GU097804-GU097811, GU097813-15, GU097817, GU097818, GU097820-32 (59)	NW Atlantic: USA (Northeast), Canada (Canadian Maritimes, Labrador, Hudson Bay)	15
S. latissima D	*Saccharina sp. ye-C*	KT315643	NW Pacific?	unpublished
	S. cichorioides	JN873239-JN873246; JQ792007-JQ792010 (12)	NW Pacific: Russia (Primorsk)	8

1. Zhang, L. *et al.* Complete mitochondrial genome of *Kjellmaniella crassifolia* (Laminariaceae , Phaeophyceae): *Laminaria* and *Saccharina* are distinct genus. *Mitochondrial DNA Part A* **27**, 4592-4594 (2016).
2. Yotsukura, N., Shimizu, T., Katayama, T. & Druehl, L. D. Mitochondrial DNA sequence variation of four *Saccharina* species (Laminariales, Phaeophyceae) growing in Japan. *J. Appl. Phycol.* **22,** 243–251 (2009).
3. Zhang, J., Wang, X., Liu, C., Jin, Y. & Liu, T. The complete mitochondrial genomes of two brown algae (Laminariales , Phaeophyceae) and phylogenetic analysis within *Laminaria*. *J. Appl. Phycol.* **25,** 1247–1253 (2013).
4. Fan, X. *et al.* Sequencing of complete mitochondrial genome of brown algal *Saccharina* sp . ye-C5. *Mitochondrial DNA Part B* **1,** 14–15 (2016).
5. Fan, X. *et al.* Sequencing of complete mitochondrial genome of brown algal *Saccharina* sp . ye-F. *Mitochondrial DNA Part A* **27,** 3693–3694 (2016).
6. Guan, Z. *et al.* Sequencing of complete mitochondrial genome of brown algal *Saccharina* sp . ye-G. *Mitochondrial DNA Part A* **27,** 2125–2126 (2016).
7. Wang, S. *et al.* Sequencing of complete mitochondrial genome of brown algal *Saccharina* sp . ye-W. *Mitochondrial DNA Part A* **27,** 3026–3027 (2016).
8. Balakirev, E. S., Krupnova, T. N. & Ayala, F. J. DNA variation in the phenotypically-diverse brown alga *Saccharina japonica*. *BMC Plant Biol.* **12,** 108 (2012).
9. Fan, X. *et al.* Sequencing of complete mitochondrial genome of brown algal *Saccharina* sp . ye-C2. *Mitochondrial DNA Part B* **1,** 16–17 (2016).
10. Xu, D. *et al.* Sequencing of complete mitochondrial genome of brown algal *Saccharina* sp. ye- C12. *Mitochondrial DNA Part B* **1,** 62-63 (2016).
11. Xu, L. *et al.* Sequencing of complete mitochondrial genome of brown algal *Saccharina* sp . ye-C6. *Mitochondrial DNA Part A* **27,** 3733–3734 (2016).
12. Kawai, H., Hanyuda, T., Ridgway, L. M. & Holser, K. Ancestral reproductive structure in basal kelp *Aureophycus aleuticus*. *Sci. Rep.* **3,** 2491 (2013).
13. Zhang, J. *et al.* Phylogeographic data revealed shallow genetic structure in the kelp *Saccharina japonica* (Laminariales, Phaeophyta). *BMC Evol. Biol.* **15,** 237 (2015).
14. McDevit, D. C. & Saunders, G. W. On the utility of DNA barcoding for species differentiation among brown macroalgae (Phaeophyceae) including a novel extraction protocol. *Phycol. Res.* **57,** 131–141 (2009).
15. McDevit, D. C. & Saunders, G. W. A DNA barcode examination of the Laminariaceae (Phaeophyceae) in Canada reveals novel biogeographical and evolutionary insights. *Phycologia* **49,** 235–248 (2010).
16. Saunders, G. W. & McDevit, D. C. A DNA barcode survey of Haida Gwaii kelp (Laminariales , Phaeophyceae) reveals novel ecological and distributional observations and *Saccharina druehlii* sp. nov. *Botany* **92,** 821–826 (2014).
17. Lane, C. E., Lindstrom, S. C. & Saunders, G. W. A molecular assessment of northeast Pacific *Alaria* species (Laminariales, Phaeophyceae) with reference to the utility of DNA barcoding. *Mol. Phylogenet. Evol.* **44,** 634–48 (2007).
18. Küpper, F. C. *et al.* Arctic marine phytobenthos of northern Baffin Island. *J. Phycol.* **52**, 532-49 (2016).
19. Wang, S. *et al.* Sequencing of complete mitochondrial genome of *Saccharina latissima* ye-C14. *Mitochondrial DNA Part A* **27,** 4037-38 (2016).
20. Silberfeld, T. *et al.* A multi-locus time-calibrated phylogeny of the brown algae (Heterokonta, Ochrophyta, Phaeophyceae): Investigating the evolutionary nature of the 'brown algal crown radiation'. *Mol. Phylogenet. Evol.* **56,** 659–74 (2010).

Table S2. Intra- and interspecific COI divergence in *Saccharina* spp. Ranges (min-max) of estimated K2P distances within (grey diagonal) and between *Saccharina* spp. (top), and within (grey diagonal) and between phylogroups of *S. latissima s. l.* (bottom).

	**sculpera (1)**	**angustata (1)**	**japonica (10)**	**bongardiana (3)**	**nigripes (6)**	**druhelii (1)**	**sessilis (8)**	**latissima sl (32)**
**S. sculpera**	NA							
**S. angustata**	5.56	NA						
**S. japonica**	5.73-6.23	4.42-4.91	**0.15-0.77**					
**S. bongardiana**	5.40-5.73	5.75-5.91	6.40-7.26	**0.15-0.30**				
**S. nigripes**	5.23-5.71	5.09-5.57	5.74-7.08	2.01-2.80 (3.27)*	**0.15-0.46 (1.07)***			
**S. druhelii**	4.57	5.40	5.06-5.56	2.64-2.96	2.16-2.48 (2.95)*	NA		
**S. sessilis**	5.40-6.07	5.56-6.24	5.89-6.74	3.60-4.08	2.96-3.60 (4.08)*	1.70-2.01	**0.15-0.77**	
**S. latissima s.l.**	4.74-5.56	5.25-6.43	4.56 (4.74)*-6.06	5.22-6.74	4.74-6.24 (6.73)*	4.72-5.55	6.22-7.41	**0.15-1.54* (1.70)**
	**latissima A (10)**	**latissima B (9)**	**latissima C (10)**	**latissima D (3)**				
**S. latissima A**	0.15-0.61* (0.92)							
**S. latissima B**	0.76-1.38* (1.54)	0.15-0.61* (0.92)						
**S. latissima C**	0.92-1.54* (1.69)	0.76-1.38* (1.70)	0.15-0.46					
**S. latissima D**	0.61-1.07* (1.23)	0.76-1.23* (1.38)	0.92-1.38	0.15-0.46				

*after removing 4 sequences (1 *S. nigripes*, 2 *S. latissima* A, 1 *S. latissima* B) with clustered mutations suggesting low quality sequenced regions (i.e. artefacts)

Table S3. Posterior distributions (mean and quantiles) of the demographic and mutation model parameters in the first hierarchical scenario.

Parameter	Mean	q(0.05)	q(0.95)
**t1 (pre-LGM)**	8.97e^4	6.93e^4	2.93e^5
**t2 (pre-LGM)**	1.97e^5	2.70e^4	4.69e^5
**µmic**	1.20e-4	1.00e-4	2.24e-4
**P**	1.93e-1	1.04e-1	2.92e-1
**SNI**	1.76e-7	1.06e-8	1.11e-6

µmic: mean mutation rate; P: shape of gamma distribution in loci mutation rate; SNI: rate of Single Nucleotide Insertion) inferred in the first hierarchal level of the ABC analyses.

Table S4. Posterior distributions (mean and quantiles) of the demographic and mutation model parameters in the second hierarchical scenario.

Parameter	Mean	q(0.05)	q(0.95)
**Admixture rate (RA)**	1.77e-1	4.73e-2	3.99e-1
**t05 (post-LGM)**	3.55e3	7.39e2	4.94e+3
**µmic**	1.97e-4	1.00e-4	2.55e-4
**P**	2.17e-1	1.04e-1	3.00e-1
**SNI**	3.67e-7	1.29e-8	2.51e-6

µmic: mean mutation rate; P: shape of gamma distribution in loci mutation rate; SNI: rate of Single Nucleotide Insertion) inferred in the second hierarchal level of the ABC analyses.

Table S5. Prior distributions for demographic and mutation parameters used in the first hierarchal level of the ABC analyses. Category of parameters: (n) effective population size, (t) time in generations. Distribution of parameters as uniform (uni) and Log-Uniform (lu), both with a min and max. Mutation model parameters (ml; microsatellite loci) as mean mutation rate(µmic), shape of gamma distribution in loci mutation rate (P) and rate of Single Nucleotide Insertion (SNI).

Parameter	Category	Distribution	Minimum	Maximum
**Ancient pop. size**	n	uni	1000	1000000
**Pacific pop. size at sampling (Pop 1)**	n	uni	1000	1000000
**NW Atlantic pop. size at sampling (Pop 2)**	n	uni	1000	1000000
**NE Atlantic pop. size at sampling (Pop 3)**	n	uni	1000	1000000
**t1 (pre-LGM)**	t	uni	10000	1000000
**t2 (pre-LGM)**	t	uni	10000	1000000
**t3 (post-LGM)**	t	uni	10	10000
**t4 (post-LGM)**	t	uni	10	10000
**µmic**	ml	uni	1×10^{-4}	1×10^{-3}
**P**	ml	uni	1×10^{-1}	3×10^{-1}
**SNI**	ml	lu	1×10^{-8}	1×10^{-5}

Table S6. Prior distributions for demographic and mutation parameters used in the second hierarchal level of the ABC analyses. Category of parameters: (n) effective population size, (t) time in generations. Distribution of parameters as uniform (uni) and Log-Uniform (lu), both with a min and max. Mutation model parameters (ml; microsatellite loci) as mean mutation rate(µmic), shape of gamma distribution in loci mutation rate (P) and rate of Single Nucleotide Insertion (SNI).

Parameter	Category	Distribution	Minimum	Maximum
**Ancient pop. Size**	n	uni	1000	1000000
**Pacific pop. size at sampling (Pop 1)**	n	uni	1000	1000000
**NW Atlantic pop. size at sampling (Pop 2)**	n	uni	1000	1000000
**Admix pop. size at sampling (Pop 3)**	n	uni	1000	1000000
**NE Atlantic pop. size at sampling (Pop 4)**	n	uni	1000	1000000
**Admixture rate (RA)**	r	uni	0.001	0.999
**t1 (pre-LGM)**	t	uni	10000	1000000
**t2 (pre-LGM)**	t	uni	10000	1000000
**t05 (post-LGM)**	t	uni	10	10000
**µmic**	ml	uni	1×10^{-4}	1×10^{-3}
**P**	ml	uni	1×10^{-1}	3×10^{-1}
**SNI**	ml	lu	1×10^{-8}	1×10^{-5}

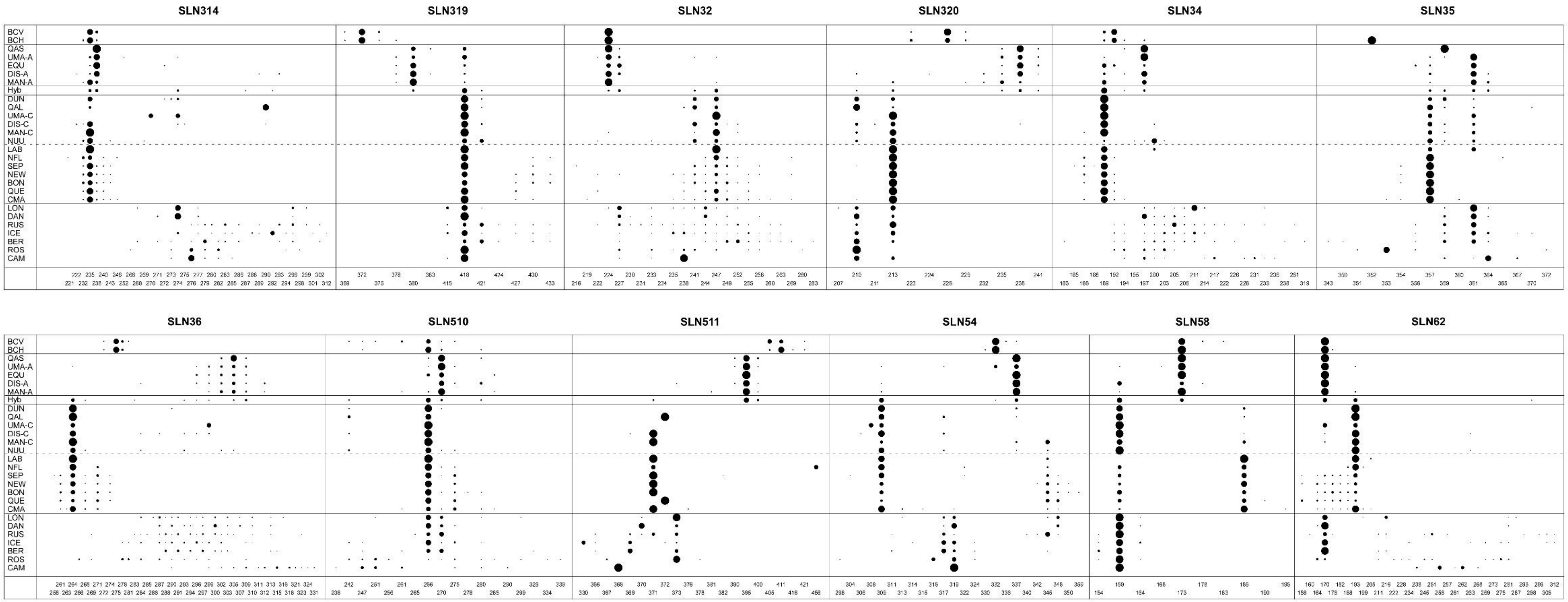

Figure S1. Microsatellite allele frequencies in each genetic population of *Saccharina latissima* s.l. The presence of an allele in a population is indicated by a circle with an area proportional to its frequency. Numbers on bottom are allele sizes (bp). Horizontal lines separate, for each genetic group, the geographical regions considered (see Table 1, also for population codes).

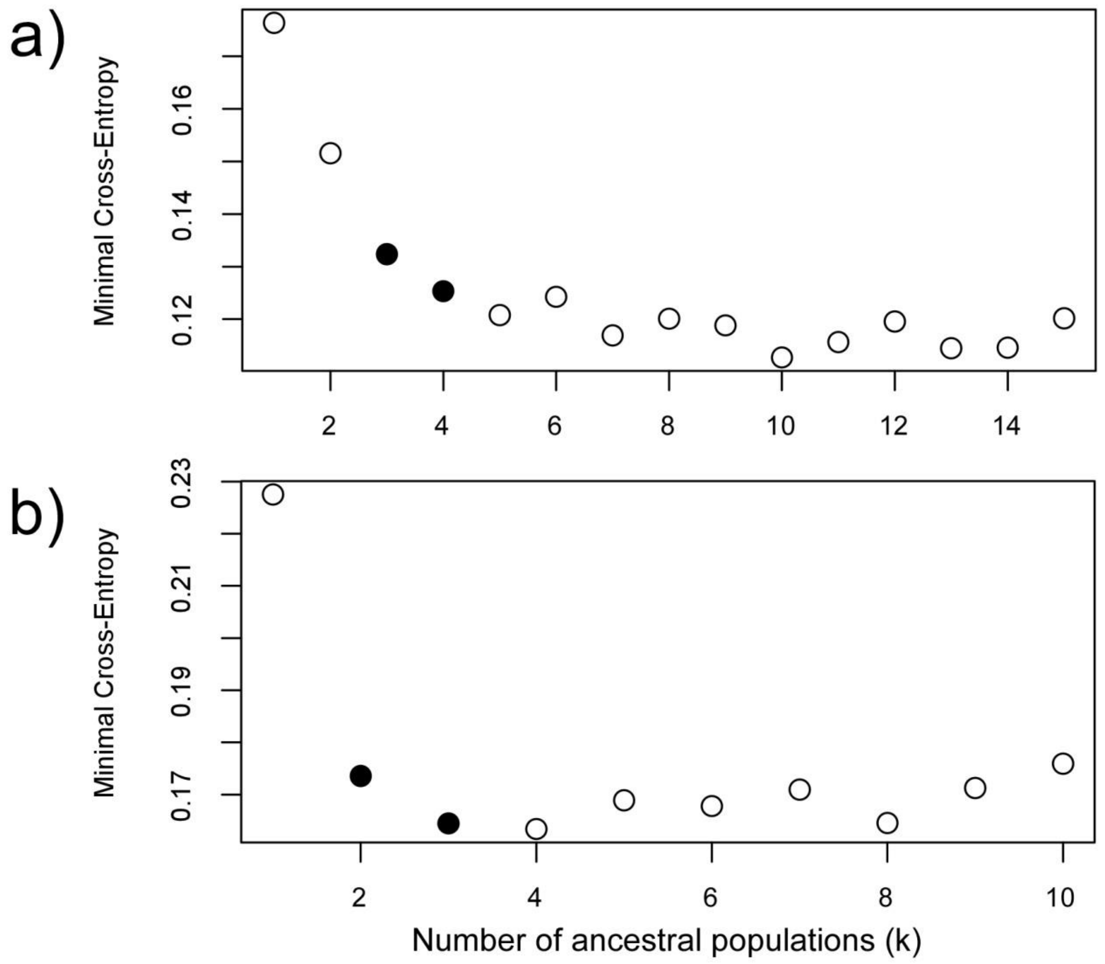

Figure S2. Genetic subdivision of *S. latissima* according to LEA analyses.

Inferred nº of genotypic clusters in **a)** the entire sampled range (NE Pacific and N Atlantic) and **b)** the NW Atlantic sympatric range of A and C phylogroups. Twenty iterations were run for each number of genetic clusters assumed (K). The most probable Ks (filled symbols) were selected based on the stabilization of the Minimal Cross-Entropy curves.

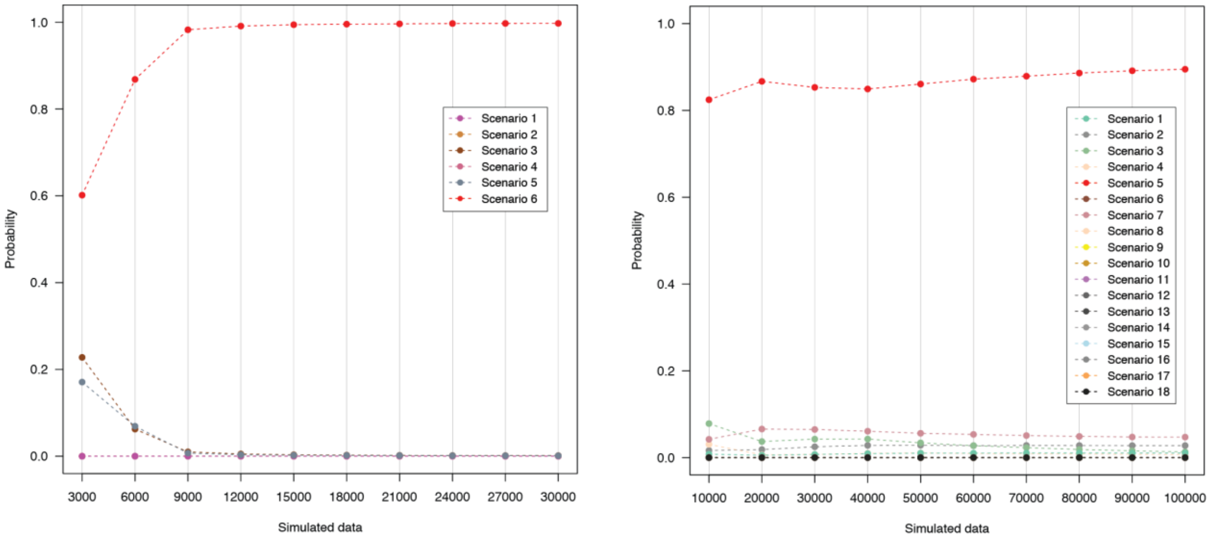

Figure S3. Logistic regression of the posterior probabilities of the tested demographic scenarios. Plots refer to the first (left) and second (right) hierarchal levels of ABC analyses, as a function of number of simulated datasets.

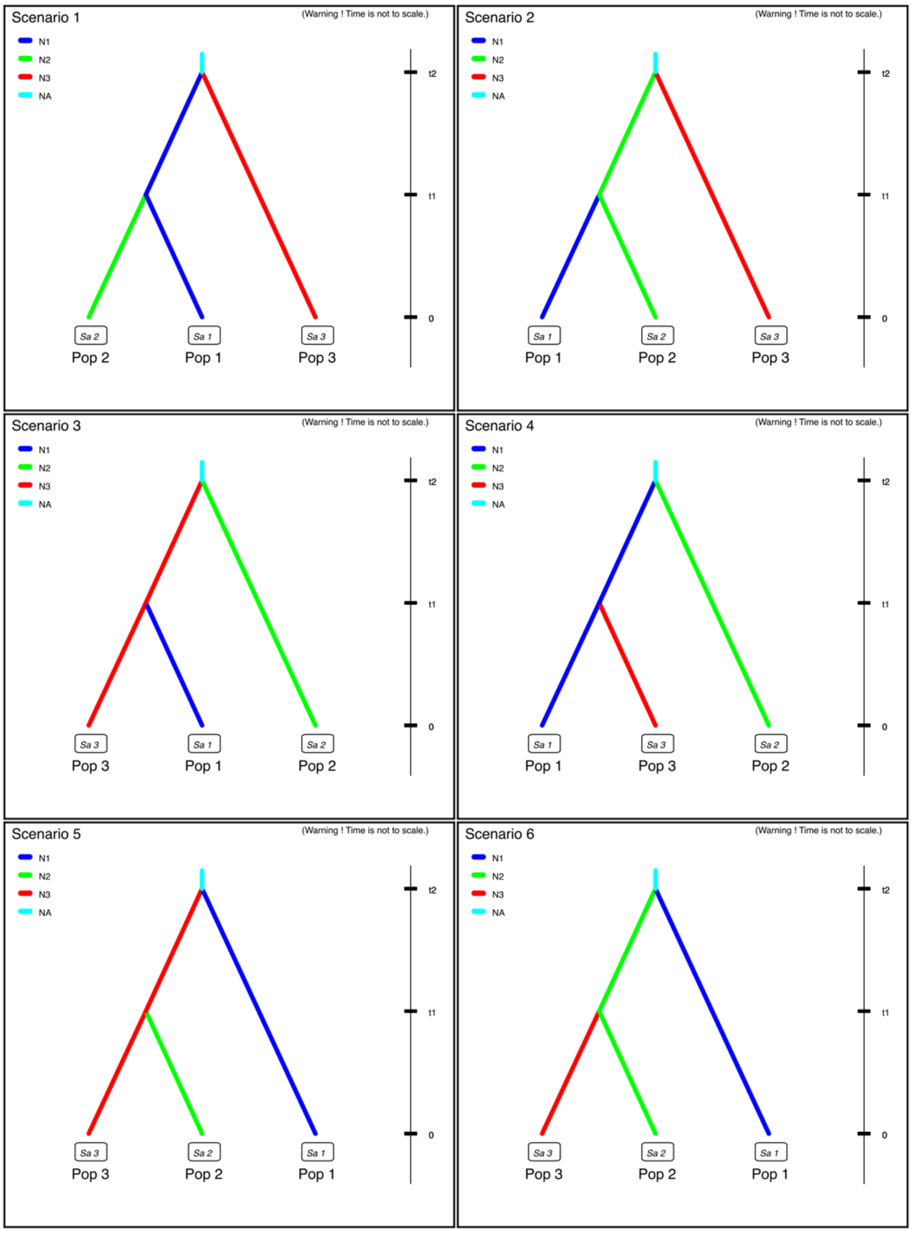

Figure S4. Scenarios compared on the first hierarchal level of ABC analyses. Three main regional (allopatric) populations of *S. latissima* were considered (Pop 1: Pacific (British Columbia), in blue; Pop 2: allopatric NW Atlantic, in green; Pop 3: NE Atlantic, in red). “A” represents the ancestral species.

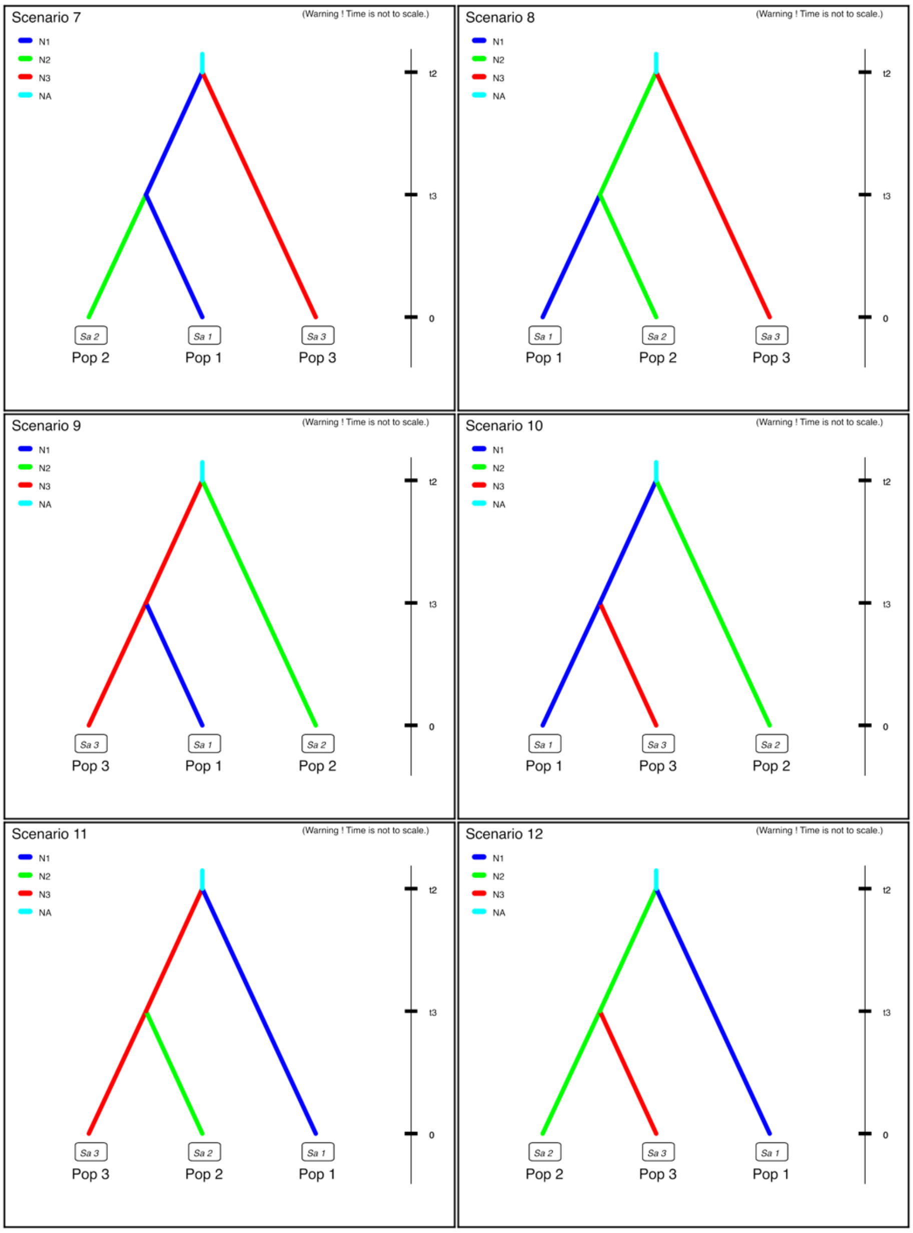

Figure S4 (cont.). Scenarios compared on the first hierarchal level of ABC analyses, performed on the three main populations of *S. latissima* (Pop 1: Pacific; Pop 2: NW Atlantic; Pop 3: NE Atlantic).

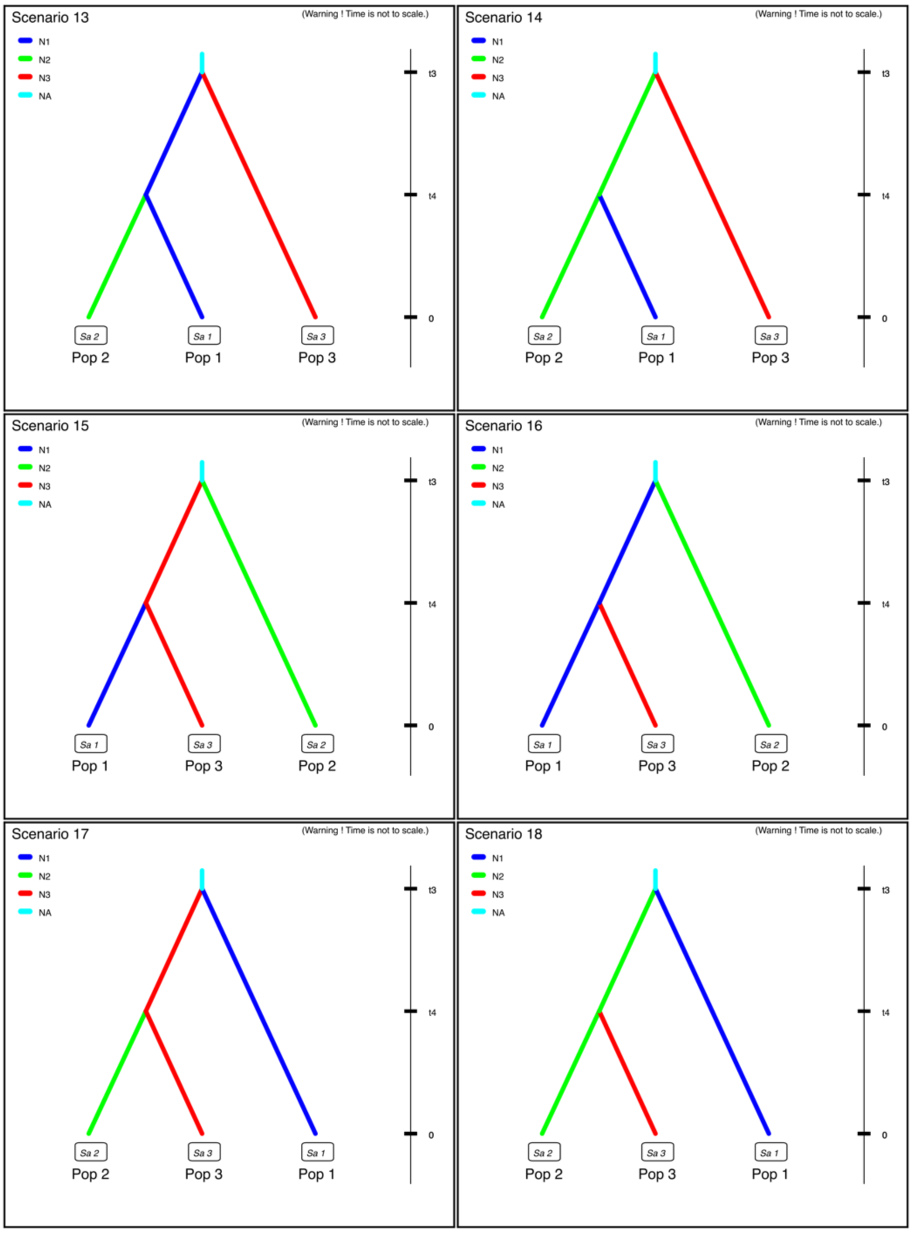

Figure S4 (cont.). Scenarios compared on the first hierarchal level of ABC analyses, performed on the three main populations of *S. latissima* (Pop 1: Pacific; Pop 2: NW Atlantic; Pop 3: NE Atlantic).

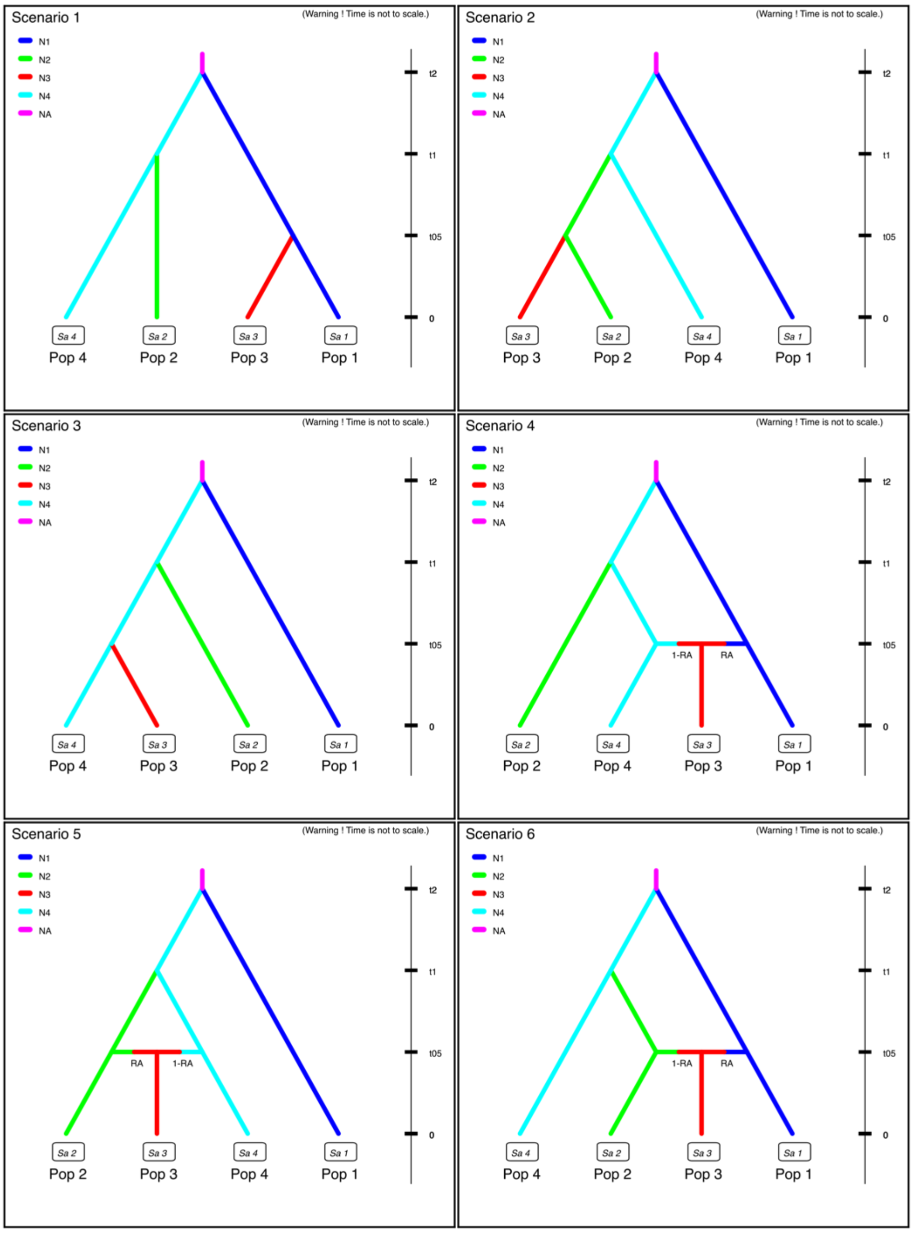

Figure S5. Scenarios compared on the second hierarchal level of ABC analyses. Four populations of *S. latissima* were considered (Pop 1: Pacific (British Columbia), in dark blue; Pop 2: allopatric NW Atlantic, in light green; Pop 3: sympatric NW Atlantic contact zone, in red; and Pop 4: NE Atlantic, in light blue). "A" represents the ancestral species.
